# Inhibition of EGFR signaling with Spautin-1 represents a novel therapeutics for prostate cancer

**DOI:** 10.1186/s13046-019-1165-4

**Published:** 2019-04-11

**Authors:** Yuning Liao, Zhiqiang Guo, Xiaohong Xia, Yuan Liu, Chuyi Huang, Lili Jiang, Xuejun Wang, Jinbao Liu, Hongbiao Huang

**Affiliations:** 1 Affiliated Cancer Hospital and institute of Guangzhou Medical University; Key Laboratory of Protein Modification and Degradation; State Key Laboratory of Respiratory Disease, School of Basic Medical Sciences, Guangzhou Medical University, Guangzhou, 511436 Guangdong China; 2 The Sixth Affiliated Hospital of Guangzhou Medical University, Qingyuan People’s Hospital, Qingyuan, 511500 Guangdong China; 3 Division of Basic Biomedical Sciences, Sanford School of Medicine of the University of South Dakota, Vermillion, SD 57069 USA

**Keywords:** Prostate cancer, EGFR, Spautin-1, Glut1, Apoptosis

## Abstract

**Background:**

Prostate cancer (PCa) remains a challenge worldwide. Due to the development of castration-resistance, traditional first-line androgen deprivation therapy (ADT) became powerlessness. Epidermal growth factor receptor (EGFR) is a well characterized therapeutic target to treat colorectal carcinoma and non-small cell lung cancer. Increasing studies have unraveled the significance of EGFR and its downstream signaling in the progression of castration-resistant PCa.

**Method:**

MTS, colony formation and Edu staining assays were used to analyze the cell proliferation of PCa cells. Flow cytometry was used to analyze PCa cell cycle distribution and cell apoptosis. Western blot was used to measure the expression of key proteins associated with cell cycle progression, apoptosis and EGFR signaling pathways. Transfection of exogenous small interfering RNA (siRNA) or plasmid was used to intervene specific gene expression. Nude mouse model was employed to test the in vivo effect of Spautin-1.

**Results:**

The current study reveals that Spautin-1, a known inhibitor of ubiquitin-specific peptidase 10 (USP10) and USP13, inhibits EGFR phosphorylation and the activation of its downstream signaling. Inhibition of EGFR signaling induced by Spautin-1 leads to cell cycle arrest and apoptosis of PCa in a USP10/USP13 independent manner. The application of Spautin-1 reduces the expression of glucose transporter 1 (Glut1) and dramatically induces cell death under glucose deprivation condition. In vivo experiments show a potent anti-tumor effect of Spautin-1 alone and in combination with Enzalutamide.

**Conclusion:**

This study demonstrates the therapeutic potential of EGFR signaling inhibition by the use of Spautin-1 for PCa treatment.

**Electronic supplementary material:**

The online version of this article (10.1186/s13046-019-1165-4) contains supplementary material, which is available to authorized users.

## Background

Prostate cancer (PCa) is the second most frequently diagnosed carcinoma among males with a high fatality rate worldwide [1]. Although the application of androgen deprivation therapy (ADT) makes a great achievement in PCa treatment, many patients are not sensitive to this treatment or inevitably progress to the castration-resistant state, which renders PCa incurable even to the present time [2, 3]. Therefore, identifying novel therapeutics for patients with ADT-insensitive PCa or castration-resistant PCa (CRPC) warranted.

Epidermal growth factor receptor (EGFR) is crucial for the development and proliferation of multiple cancers, including colorectal carcinoma, non-small cell lung cancer, and PCa [4–6]. Hence, EGFR stands out as a target to treat these cancers. Recent studies have also been revealed that aberrant activity of EGFR may drive the development of CRPC, possibly due to the deprivation of androgen signaling [7, 8]. EGFR is a well-documented member of the transmembrane receptor tyrosine kinase family [9]. The binding of ligands, such as EGF, TNFα, etc. leads to the transphosphorylation of EGFRs, which provides a docking site for SRC homology 2 (SH2) domain-containing signaling proteins [10]. Activation of these molecules subsequently regulate their downstream effectors, including mitogen-activated protein kinase (MAPK) and phosphoinositide-3-kinase (PI3K)/−AKT pathways [11, 12], and thereby mediating multiple physiological and pathological processes, including cell cycle progression and cell survival and invasion that promote the development of multiple cancers [13–15].

Spautin-1 acts as an inhibitor of ubiquitin-specific peptidase 10 (USP10) and USP13 via promoting the degradation of Vps34, and has been documented as an autophagy inhibitor [16]. Subsequent studies showed that Spautin-1 enhances the anticancer activity of targeted-therapy or traditional chemotherapy via autophagy inhibition in several cancers [17–19]. Recent study also showed that Spautin-1 activates c-Jun N-terminal kinase (JNK) and thereby inducing immunogenic cell death [20]. These findings provided alternative evidence that Spautin-1 may serve as a novel agent with potential therapeutic implication for certain cancers. However, whether Spautin-1 could be used as a therapeutic agent to treat PCa or not and its working mechanism remain unknown.

In the current study, we report that Spautin-1 significantly suppressed the growth of PCa by arresting cell cycle progression and triggering apoptosis. Mechanistically, Spautin-1 inhibits EGFR and its downstream signaling pathways, leading to activation of the MKK4/JNK/Bax axis and inactivation of the MEK1/2/ERK/Cyclin D1 axis. The downregulation of Glut1 induced by Spautin-1 notably increased cell death in glucose deprivation condition. Moreover, the application of Spautin-1 surprisingly enhanced the anti-cancer activity of Enzalutamide both in vitro and in vivo. Together, these findings reveal that Spautin-1 could be developed as a clinically available compound against CRPC via inhibition of EGFR signaling pathways.

## Methods

### Materials

Spautin-1(S7888), 3-methyladanine (3-MA, S2767), Z-VAD-FMK (S7023), SP600125 (S1460), SB230580 (S1076), LY3214996 (S8534), Gefitinib (S1025) and Enzalutamide (MDV3100, S1250) were purchased from Sellectchem (Houston, TX, USA). SKP2-C25 (M60136) was obtained from Xcessbio Biosciences, Inc. (San Diego, CA). USP10 (sc-76,811), USP13 (sc-76,815) and Glut1 (sc-35,493) siRNAs were purchased from Santa Cruz Biotechnology (Santa Cruz, CA, USA). Antibodies: anti-Glut1 (ab652), anti-USP13 (ab109264) (Abcam, Cambridge, MA); anti-GAPDH (BS60630), anti-Ki67 (BS1454) (Bioworld Technology, Inc., Louis Park, MN, USA); anti-USP10(#8501), anti-SKP2(#2652), anti-p27(#3686), anti-CDK4(#12790), anti-CDK2(#2546), anti-CyclinD1(#2978), anti-p15(#4822), anti-p21(#2947), anti-PARP(#9532), anti-Bim(#2933), anti-Bax(#14796), anti-Bcl-2(#15071), anti-activated Caspase-3(#9664), anti-phospho-JNK (#9255), anti-JNK(#9252), anti-phospho-ERK1/2(#4370), anti-ERK1/2(#4695), anti-phospho-p38(#4511), anti-p38(#8690), anti-phospho-MKK4(#4514), anti-MKK4(#9152), anti-phospho-MEK1/2 (#9154), anti-MEK1/2(#4694), anti-phospho-EGFR (Tyr1173)(#4407), anti-phospho-EGFR (Tyr1068)(#3777), anti-EGFR(#2085) (Cell Signaling Technology, Beverly, MA, USA).

### Cell culture

The following cell lines were purchased from ATCC (American Type Culture Collection, Manassas, VA, USA): WPMY-1, 22Rv1, LNCaP, PC3, C4–2 and DU145. WPMY-1 cells were grown in DMEM supplemented with 10% FBS. 22Rv1, LNCaP and C4–2 cells were grown in RPMI 1640 supplemented with 10% FBS. PC3 and DU145 cells were grown in Hyclone DMEM/F-12 supplemented with 10% FBS. Cultured cells were maintained at 37 °C and 5% CO2.

### Cell viability assay

MTS (catalog no. G111) was purchased from Promega Corporation (Madison, WI, USA) and used to test cell viability as we reported [21]. Briefly, exponentially growing PCa cells were seeded at 2000 cells/well in a 96-well plate. After incubation for 24 h, cells were treated with increasing doses of Spautin-1 for 24, 48 or 72 h. 20 μl MTS reagent was directly added to each well and cells were incubated for additional 3 h. The absorbance of optical density (OD) was measured with a microplate reader (Sunrise, Tecan, Mannedorf, Switzerland) at wavelength 490 nm.

### Clonogenic assay

This assay was performed as previously described [22, 23]. PCa cells were exposed to Spautin-1 at 10 μM or control solvent for 24 h. Cells post Spautin-1 or control solvent treatment were then digested, resuspended and seeded in 60 mm dishes supplemented with 10% FBS medium. Cells were then cultured in an atmosphere of 5% CO2 for 2 weeks. For crystal violet staining of colonies, cells were fixed with 4% paraformaldehyde for 15 min, then washed with PBS twice, followed by crystal violet solution incubation for 5 min. Colonies > 60 μm were counted from three independent experiments.

### Cell cycle and proliferation assay

For cell cycle assay, 22Rv1, PC3 and LNCaP cells treated with Spautin-1 or SKP2-C25 treatment for 24 h were digested, harvested and washed with cold PBS twice. After discarding supernatant, cells were resuspended with 500 μl PBS and 2 ml 70% ethanol at 4 °C overnight. And then the cells were washed with 4 °C PBS twice again, followed by incubation with PI (50 μg/ml) (Keygen, Nanjing, China), RNase A (100 μg/ml) and 0.2% Triton X-100 mixtures for 30 min at 4 °C in dark. Cell cycle distributions of each group were ultimately analyzed with flow cytometry. For cell proliferation assay, PC3 cells post Spautin-1, SKP2-C25, 3-MA, USP10 siRNAs or USP13 siRNAs treatment were incubated with EdU (5-ethynyl-2′-deoxyuridine, Ribobio, Guangzhou, China) at 50 μM for 2 h. Apollo was used to probe EdU. DAPI was used to indicate the nucleus. Nuclear EdU level indicates the activity of DNA replication and cell proliferation.

### Cell death assay

Analysis of cell death was performed as we described [24, 25]. Briefly, PCa cells post Spautin-1, USP10 KD or USP13KD treatment were digested, collected, and washed with cold PBS for three times. After discarding supernatant, cells were resuspended with 500 μl annexin V-FITC binding buffer, 5 μl annexin V-FITC, and 5 μl PI mixture (Keygen, Nanjing, China) in each group. Cells were then incubated with annexin V-FITC/PI mixture for 30 min, followed by the analysis of stained cells using flow cytometry.

### SiRNA and plasmid transfection

Transfection of nucleic acids was performed as we reported [22, 24]. PCa cells were randomly seeded in 60 mm dishes or 96-well plates for 24 h. For siRNAs transfection, RPMI opti-MEM (Gibco), lipofectamine RNAiMax (Invitrogen) reagent and siRNAs (Santa Cruz, CA) targeting human USP10/USP13/Glut1 siRNAs (Santa Cruz, CA), or control siRNAs (non-specific sequences) mixtures were prepared, respectively. After incubation for 15 min, the mixtures was added in each group. Cells were cultured for 48 h and 72 h for further analysis. For plasmid transfection, RPMI opti-MEM (Gibco), lipofectamine 2000 (Invitrogen) reagent and eukaryotic expression vector pcDNA3.1(+)-SKP2-HA (NM005983), pENTER-CDK2-HA (NM_001798.4), pENTER-CyclinD1-FLAG (NM_053056.2), pEGFP-N1-Glut1-HA (NM_006516) plasmids or their control vector mixtures were prepared respectively. After incubation for 15 min, the mixtures was added in each group and cultured for 48 h and 72 h for further analysis. Fresh medium was replaced appropriately after transfection for 6 h.

### Western blot

Western blot analysis was performed as we described [26, 27]. In brief, equal amounts of total proteins extracted from PCa cells exposed to Spautin-1 or USP10/USP13/Glut1 siRNAs were fractionated using 12% SDS–PAGE. The separated proteins on SDS–PAGE were subsequently transferred to polyvinylidene difluoride (PVDF) membranes. The blots were blocked with 5% milk for 1 h. Primary antibodies and horseradish peroxidase (HRP)-conjugated secondary antibodies were each incubated for 1 h. The bounded secondary antibodies were reacted to the ECL detection reagents and exposed to X-ray films (Kodak, Japan).

### Immunofluorescence

This assay was performed as we described [28, 29]. Briefly, PCa cells post Spautin-1 treatment were fixed with 4% paraformaldehyde for 15 min. 0.5% Triton-X was used to permeabilize cells for 5 min. 5% BSA (bovine serum albumin, Sigma) was used to block for 30 min. And then the cells were incubated with Phospho-JNK, Phospho-ERK or Glut1 primary antibodies overnight at 4 °C. Next the cells were incubated with anti-mouse IgG H&L (Alexa Fluor 488, Abcam) or anti-rabbit IgG-cy3 (Bioworld) secondary antibodies for 1 h. DAPI (4′,6-diamidino-2-phenylindole, Abcam) was used to indicate the nucleus. Images were acquired using an Olympus fluorescence microscope with 400× magnification.

### Nude mouse xenograft model

Nude Balb/c mice were bred and housed at the animal center of Guangzhou Medical University in accordance with ethical treatment of animals. The xenograft models were prepared as previously reported [30, 31]. Briefly, 2 × 10^6^ 22Rv1 or PC3 cells were inoculated subcutaneously on the flanks of 5- to 6-week-old male nude mice. After inoculation for 3 days, the mice successfully bearing xenografts were bred and treated with either Spautin-1 (20 mg/kg/day, i.p.), Enzalutamide (25 mg/kg/day, p.o.) or vehicle for a total of 30 days. The size of xenografts was measured and calculated as previously reported [30].

### Immunohistochemical staining

Xenografts were fixed with formalin and sectioned according to standard techniques. MaxVision kit (Maixin Biol) was used to immunostain xenograft sections (4 μm) according to a previous report [30]. Primary antibodies were against Ki67. MaxVisionTM reagents were added one by one on the slide in 50 μl according to the instruction. Color was developed with 0.05% DAB and 0.03% H2O2 in 50 mM Tris-HCl (pH 7.6), and the slides were counterstained with hematoxylin.

### Statistical analysis

Data are presented as mean ± SD from three independent experiments where applicable. To determine statistical probabilities, unpaired Student’s t test or one way ANOVA is used where appropriate. Statistical analysis was performed by GraphPad Prism5.0 software (GraphPad Software) and SPSS 16.0. A *P* value of < 0.05 was considered statistically significant.

## Results

### Spautin-1 suppressed the proliferation of PCa cells independent of autophagy inhibition and the USP10/USP13-SKP2-p27 axis

**Fig. 1:**
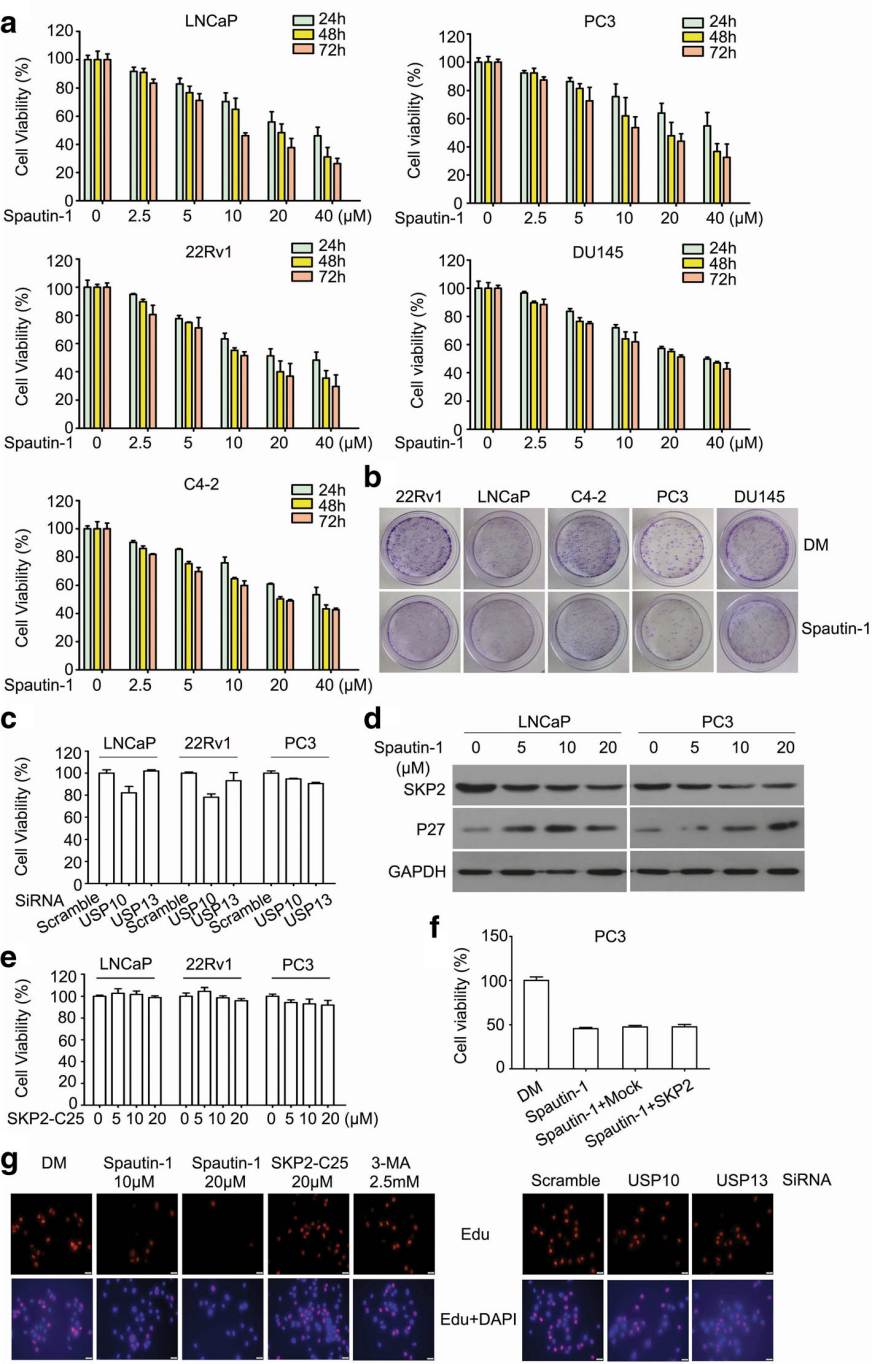
Spautin-1 suppresses the proliferation of PCa independent of USP10 and USP13. **a** Cell viability assay was performed in LNCaP, 22Rv1, C4–2, PC3 and DU145 PCa cells post various concentrations of Spautin-1 treatment for indicated hours. **b** Colony formation assay was performed in PCa cells post Spautin-1 (10 μM) treatment for two weeks. DM, DMSO. **c** Cell viability assay was performed in LNCaP, 22Rv1 and PC3 treated with control siRNA, USP10 siRNA, or USP13 siRNA for 72 h. **d** Western blot analysis was used to detect the expression of SKP2 and p27 in LNCaP and PC3 cells treated with Spautin-1 for 24 h. GAPDH was used as a loading control. **e** Cell viability assay was performed in LNCaP, 22Rv1 and PC3 treated with SKP2-C25 for 48 h. **f** Cell viability assay was performed in PC3 cells pre-transfected with control vector or SKP2 plasmid for 24 h before Spautin-1 (20 μM) treatment for 48 h. **g** Edu staining assay was performed to detect the proliferation ability of PC3 cells treated with Spautin-1, SKP2-C25, or 3-MA treatment for 24 h, or 48 h after subject to USP10 knockdown (KD) and USP13 KD. The staining of Edu in the nucleus indicates the cell in proliferation

To determine the anti-tumor effect of Spautin-1 on PCa, five PCa cell lines, including LNCaP, 22Rv1, C4–2, PC3 and DU145, and normal prostate cell line WPMY-1 were employed in this study. Cell viability assay was used to detect the proliferation of PCa cells in the presence of escalating doses of Spautin-1. We found that Spautin-1 remarkably suppressed the cell viability of prostate cancer cells within three days (Fig. 1a), but only moderately inhibits the cell viability of WPMY-1 cells (Additional file 1: Figure S1a). We further determined the colony formation ability of these cell lines after Spautin-1 treatment for 24 h and found that Spautin-1 also remarkably suppressed the colony formation among these cells, regardless of AR expression status (Fig. 1b and Additional file 1: Figure S1b). Therefore, we submit that Spautin-1 has a potent anti-CPRC activity. In consideration of the USP10/USP13 inhibition effect of Spautin-1 [16], we determined whether USP10/USP13 was involved in the proliferation inhibition of Spautin-1 on PCa cells. Cell viability assay was performed on PCa cells after the cells had been subject to USP10/USP13 siRNAs treatment for 72 h. The effects of USP10/USP13 knockdown (KD) were verified (Additional file 1: Figure S1c). We found that both USP10 KD and USP13 KD did not suppress the cell viability of PCa cells (Fig. 1c). Our previous study has showed that the USP10-SKP2-p27 axis mediates Spautin-1 induced cell cycle arrest in Chronic Myeloid Leukemia. We therefore further determined whether this is also true to PCa. Likewise, Spautin-1 reduced the protein level of SKP2 and up-regulated the expression of p27 in LNCaP and PC3 cells (Fig. 1d). But surprisingly, inhibition of SKP2 with SKP2-C25 did not significantly suppressed the cell viability of PCa cells (Fig. 1e). Additionally, overexpression of SKP2 in PC3 cells failed to rescue Spautin-1 induced cell viability suppression (Fig. 1f). The effects of SKP2 overexpression were verified in Additional file 1: Figure S1d. We further detected the proliferation ability of PCa cells treated with Spautin-1, SKP2-C25, 3-MA (another autophagy inhibitor), USP10 siRNAs, or USP13 siRNAs, using the Edu staining assay. The effects of USP10/USP13 KD in PC3 cells were verified (Additional file 1: Figure S1e). We found that only Spautin-1 discernibly inhibited the proliferation ability of PCa cells (Fig. 1g and Additional file 1: Figure S1f). These findings collectively suggest that proliferation inhibition of PCa by Spautin-1 is through a novel mechanism independent of autophagy inhibition and the USP10/USP13-SKP2-p27 axis.

### Spautin-1 arrested the G0/G1 to S phase transition via decreasing cyclin D1 expression

**Fig. 2:**
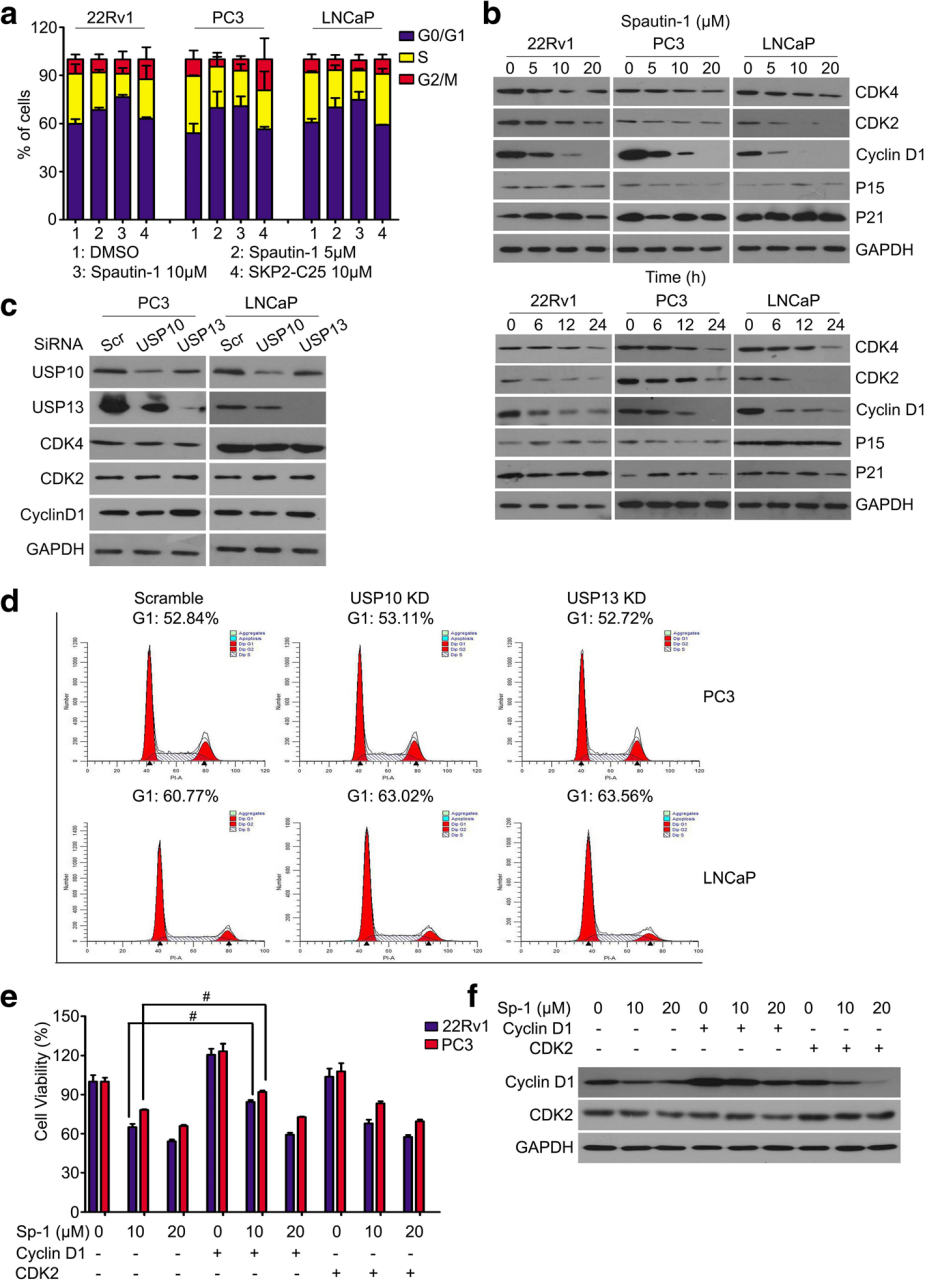
Spautin-1 induces G0/G1 phase arrest via down-regulating Cyclin D1 in PCa cells. **a** Fluorescence-activated cell sorting analysis (FACS) was performed to analyze cell cycle distributions of PCa cells exposed to Spautin-1 or SKP2-C25 for 24 h. A summary of cell cycle distributions was shown from three independent experiments. **b** Western blot analysis was performed to detect the expression of CDK4, CDK2, Cyclin D1, P15 and P21 in PCa cells exposed to various doses of Spautin-1 (0, 5, 10, 20 μM) for 24 h (left), or Spautin-1 (10 μM) at various lengths of time (Right). **c** Western blot analysis was performed to detect the expression of CDK4, CDK2, Cyclin D1, USP10 and USP13 in PCa cells exposed to USP10 siRNA or USP13 siRNA for 48 h. **d** FACS was performed to analyze cell cycle distributions of PCa cells exposed to USP10 siRNA or USP13 siRNA for 48 h. **e** 22Rv1 and PC3 cells were transfected with HA-Cyclin D1 and/or FLAG-CDK2 for 48 h. Cell viability analysis was performed on the above cells exposed to Spautin-1 for 24 h. ^#^*P*<0.05. **f** Western blot of CDK2 and Cyclin D1 to verified the overexpression in PC3 cells. Sp-1: Spautin-1

Rapid cell cycle progression is a hallmark of numerous cancers. To determine whether Spautin-1 impedes cell cycle progression of PCa, we measured cell cycle distributions using flow cytometry the cells were treated with Spautin-1 or SKP2-C25 for 24 h. We found that Spautin-1, but not SKP2-C25, significantly arrested cell cycle progression at the G0/G1 phase in 22Rv1, LNCaP and PC3 cells (Fig. 2a). To further determine the change of G0/G1 phase to S phase transition-associated checkpoint proteins, western blot analysis was used to detect the expression of CDK4, CDK2, Cyclin D1, p15 and p21 in PCa cells post Spautin-1 treatment. We found that Spautin-1 decreased the expression of CDK4, CDK2 and Cyclin D1 in a dose- and time- dependent manner but did not regularly affect the expression of p15 and p21 (Fig. 2b). Spautin-1 is a potent inhibitor of USP10/USP13; we therefore asked whether USP10/USP13 KD would decrease the expression of these molecules and induce cell cycle arrest in PCa cells. Western blot analysis was used for detecting the expression of CDK4, CDK2 and Cyclin D1 and flow cytometry for determining cell cycle distributions. We found that neither USP10 KD nor USP13 KD notably decreased the expression of CDK4, CDK2 and Cyclin D1, or changed the cell cycle distributions of PCa cells (Fig. 2c and d). These results indicate that Spautin-1 induces a USP10/USP13-SKP2-p27-independent G0/G1 phase arrest. Additionally, as shown in Fig. 2b, the decrease of CDK2 and Cyclin D1 occurred earlier and was more remarkable than the decrease of CDK4 in PCa cells post Spautin-1 treatment. To further determine the key protein mediated Spautin-1 induced cell cycle arrest, HA-Cyclin D1 plasmid or FLAG-CDK2 plasmids were transfected into PCa cells 48 h before Spautin-1 treatment. Interestingly, overexpression of Cyclin D1, but not CDK2, was able to rescue Spautin-1 (at 10 μM) induced proliferation inhibition in PCa cells. However, the forced expression of Cyclin D1 failed to reverse the proliferation inhibition induced by high dose of Spautin-1 (at 20 μM) (Fig. 2e). The effects of CDK2 or Cyclin D1 overexpression were verified in Fig. 2f. These findings collectively suggest that Cyclin D1 is the key regulator mediating Spautin-1 induced cell cycle arrest, while high dose of Spautin-1 can exhibit additional anti-cancer effects beyond G0/G1 phase arrest.

### Spautin-1 in high dose induced caspases-dependent apoptosis of PCa cells

**Fig. 3:**
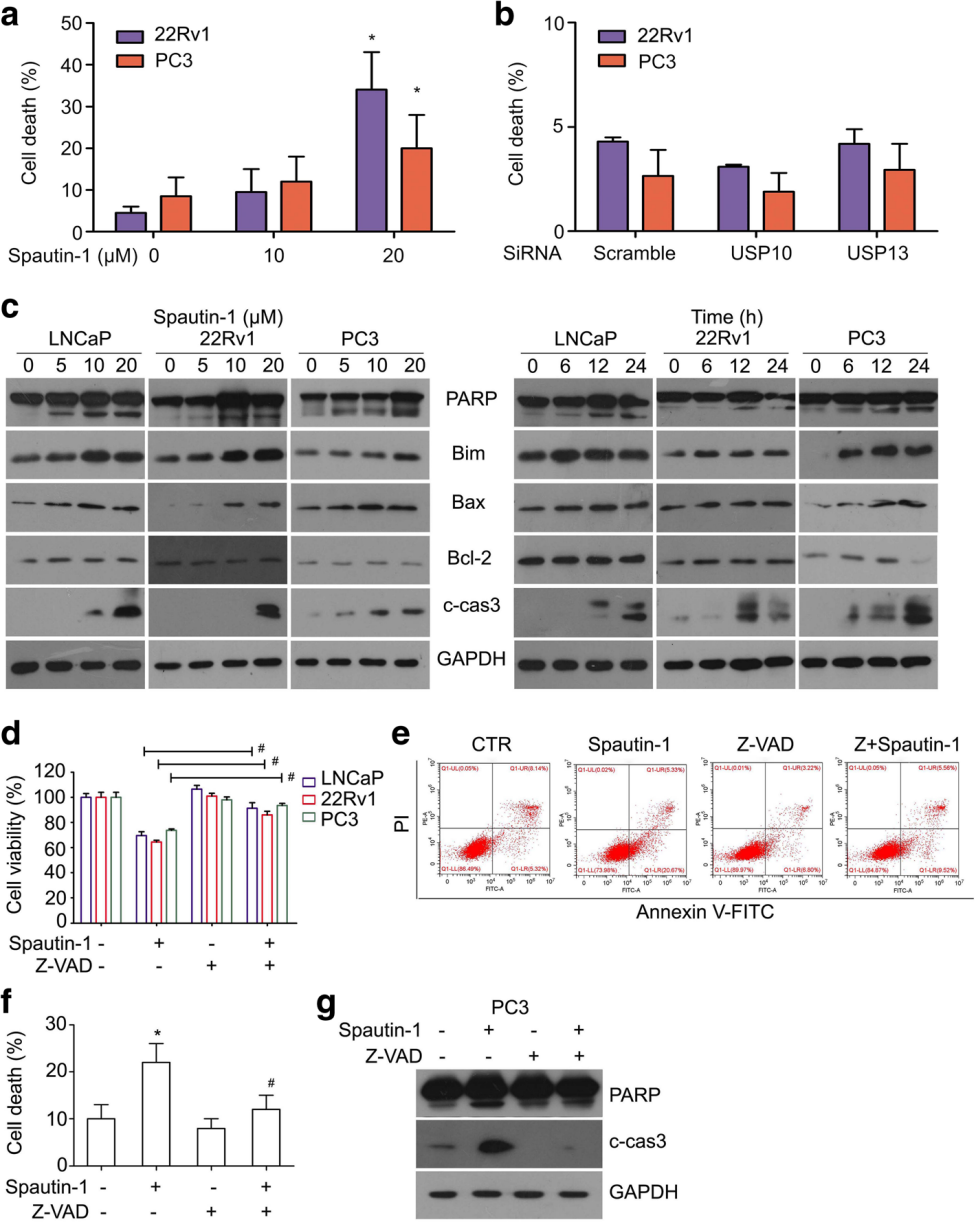
High dose of Spautin-1 triggers caspase-dependent apoptosis in PCa cells. **a** Flow cytometry analysis following Annexin V-FITC and PI staining was performed on PCa cells exposed to Spautin-1 for 24 h, or **b** subject to USP10 KD and USP13 KD for 48 h. Data of three independent repeats are summarized. ^*^P<0.05 versus control treatment. **c** Western blot analysis was performed to detect the expression of PARP, Bim, Bax, Bcl-2 and C-Cas3 (cleaved caspase3) in PCa cells exposed to various doses of Spautin-1 (0, 5, 10, 20 μM) for 24 h (left), or Spautin-1 (20 μM) at various lengths of time (Right). **d** Cell viability analysis was performed on PCa cells exposed to Spautin-1 (20 μM) in the presence or absence of Z-VAD-FMK (50 μM). ^#^*P<*0.05. **e** Apoptosis assay was performed on PC3 cells exposed to Spautin-1 (20 μM) in the presence or absence of Z-VAD-FMK (50 μM). **f** Shown are pooled data from three independent experiments. ^*^P<0.05 versus control treatment; ^#^*P<*0.05 versus Spautin-1 treatment. **g** Western blot analysis was performed to detect the expression of PARP and C-Cas3 in PC3 cells subject to Spautin-1 (20 μM) treatment in the presence or absence of Z-VAD-FMK (50 μM)

To further determine whether higher dose of Spautin-1 induces cell death of PCa cells, flow cytometry following Annexin V-FITC/PI staining was used to detect dead cells of PCa post Spautin-1 (0, 10, 20 μM) treatment. We found that Spautin-1 at 10 μM induced a modest amount of cell death, while it at 20 μM significantly increased cell death of PCa cells (Fig. 3a and Additional file 1: Figure S2a). We also determined whether USP10/USP13 KD induces cell death of PCa cells and found that neither USP10 KD nor USP13 KD noticeably induced cell death of these cells (Fig. 3b). We further detected the change of cell death-associated proteins post Spautin-1 treatment using western blot analysis and found that Spautin-1 dose- and time- dependently increased the expression of pro-apoptotic proteins Bim, Bax, cleaved caspase 3, and PARP cleavage but did not discernibly affect the expression of Bcl-2 (Fig. 3c). To further determine whether caspases are critical to Spautin-1 induced apoptosis, MTS assay (Fig. 3d), flow cytometry (Fig. 3e, f and Additional file 1: Figure S2b, c) and western blot analysis (Fig. 3g) were used to detect the cell viability, cell death and the expression of PARP cleavage and cleaved-Caspase 3, respectively, in PCa cells post Spautin-1 treatment in the presence or absence of Z-VAD-FMK, a pan caspase inhibitor. We found that, Z-VAD-FMK significantly reversed Spautin-1 induced cell viability inhibition, cell death, PARP cleavage, and the expression of cleaved caspase 3. Collectively, these findings suggest that Spautin-1, at 20 μM, induces caspase-dependent apoptosis in PCa cells.

### JNK and ERK mediated Spautin-1-induced growth inhibition

**Fig. 4:**
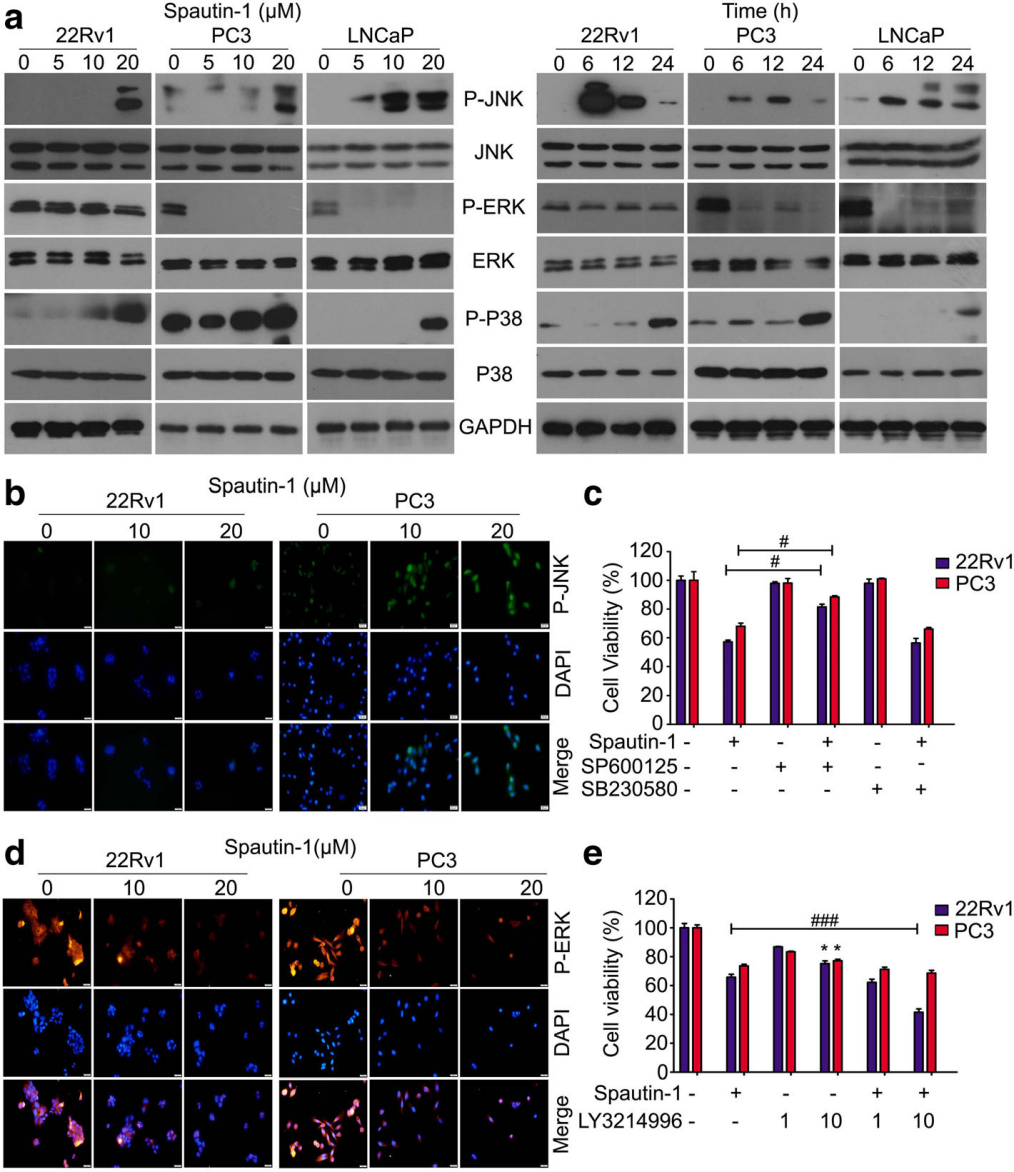
JNK and ERK mediate Spautin-1-induced growth inhibition. **a** Western blot analysis was performed to detect the expression of phosphor-JNK, JNK, phosphorylated ERK (P-ERK), ERK, P-P38 and P38 in PCa cells exposed to various doses of Spautin-1 (0, 5, 10, 20 μM) for 24 h (left), or Spautin-1 (20 μM) at various time points (Right). **b** Immunofluorescence microscopy was performed to detect the expression and subcellular location of phospho-JNK and **d** phospho-ERK in 22Rv1 and PC3 cells exposed to Spautin-1 for 6 h. Scale bars represent 50 μm. **c** Cell viability assay was performed on PCa cells exposed to Spautin-1 (20 μM) in the presence or absence of SP600125 or SB230580 for 24 h. **e** Cell viability assay was performed on PCa cells exposed to Spautin-1 (20 μM) in the presence or absence of LY3214996 for 24 h

MAPKs, including ERK (extracellular signal-regulated kinase), JNK and p38 MAPK, play a critical and complex role in the regulation of cell survival and growth in carcinomas [32, 33]. This study further determined whether Spautin-1 would affect the function of MAPKs. We found that high dose of Spautin-1 (20 μM) notably increased the expression of Phospho-JNK and Phospho-p38 in PCa cells, indicative of JNK and p38 activation by the Spautin-1 treatment. The JNK activation could be observed as early as 6 h after the initiation of Spautin-1 treatment but the activation of p38 by Spautin-1 treatment occurred between 12 h and 24 h. Additionally, we found that various doses of Spautin-1 notably decreased the expression of phospho-ERK in PC3 cells, while it moderately inhibits phospho-ERK in 22Rv1 cells, and these effects occurred at 6 h after Spautin-1 treatment, suggesting that Spautin-1 mainly affects JNK and ERK, but not p38 (Fig. 4a). Immunofluorescence microscopy also showed that Spautin-1 up-regulated the phospho-JNK level and reduced the phospho-ERK level at 6 h (Fig. 4b, d and Additional file 1: Figure S3a, b). To further decipher whether MAPKs mediate Spautin-1 induction of growth inhibition, cell viability assay was performed on PCa cells post Spautin-1 treatment in the presence or absence of SP600125 (JNK inhibitor), SB230580(p38 inhibitor), or LY3214996 (ERK inhibitor). We found that JNK inhibitor SP600125, but not SB230580, significantly reversed the cell viability inhibition induced by Spautin-1 (Fig. 4c). Additionally, ERK inhibitor LY3214996, at 10 μM, notably inhibited the proliferation of PCa cells. The application of LY3214996 enhanced Spautin-1 sensitivity in 22Rv1 cells, but not in PC3 cells (Fig. 4e). This differential effect may be caused by the expression of phospho-ERK. As showed in Fig. 4a, phospho-ERK in PC3 cells was nearly completely abolished by Spautin-1 treatment alone but this was not the case in 22Rv1 cells. Collectively, these results demonstrate that JNK and ERK MAPKs mediate the growth suppression by Spautin-1.

### Spautin-1 inhibited EGFR activation of PCa cells

**Fig. 5:**
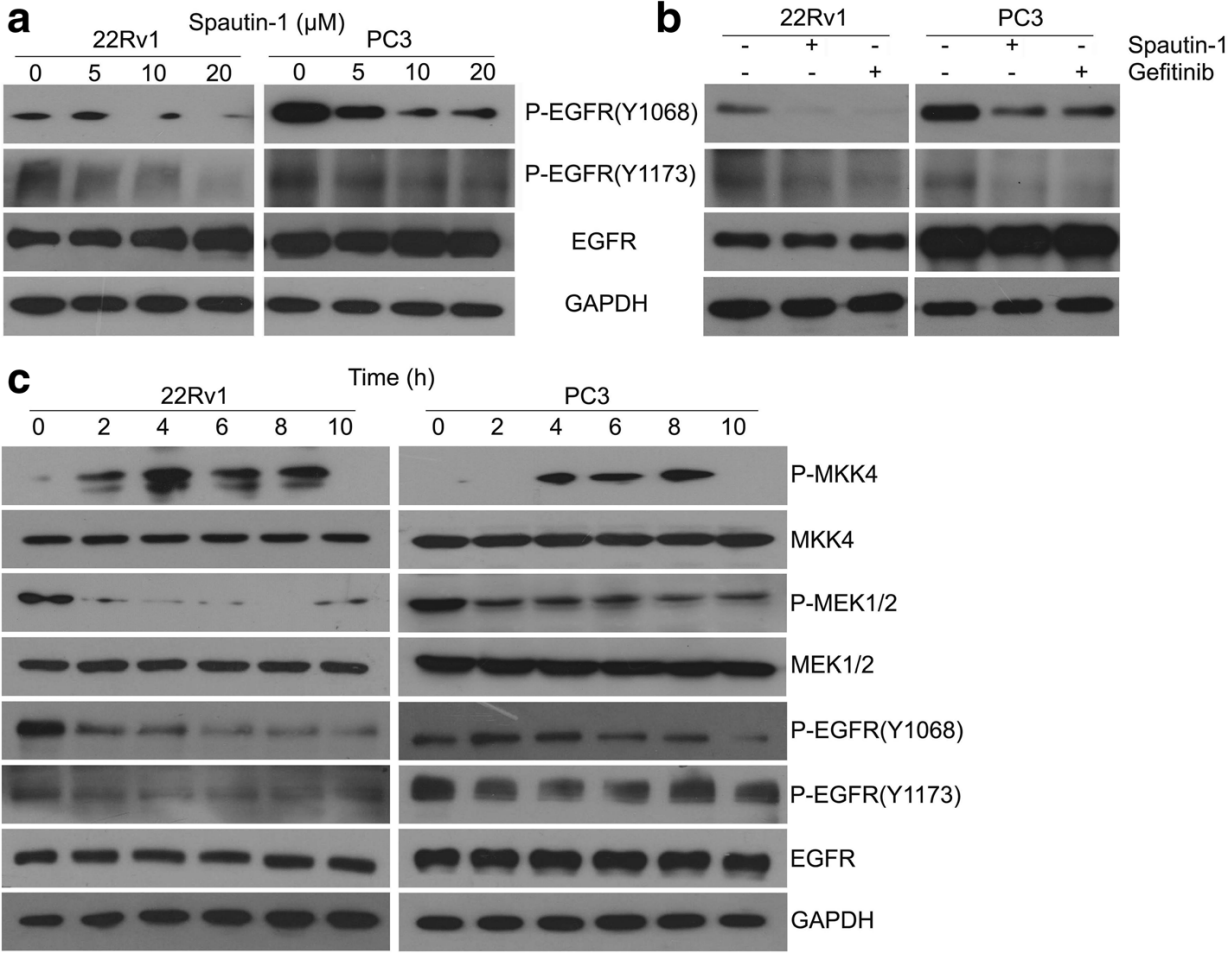
Spautin-1 inhibits EGFR signaling pathways in PCa cells. **a** Western blot analysis was performed to detect the expression of P-EGFR (Y1068), P-EGFR (Y1173) and pan-EGFR in 22Rv1 and PC3 cells treated with Spautin-1 for 24 h. **b** The expression of P-EGFR (Y1068), P-EGFR (Y1173) and pan-EGFR in 22Rv1 and PC3 cells exposed to Spautin-1 (20 μM) or Gefitinib (2 μM) for 24 h were detected using western blot. **c** Western blot analysis was performed to detect the expression of P-MKK4, MKK4, P-MEK1/2, MEK1/2 P-EGFR(Y1068), P-EGFR(Y1173) and pan-EGFR in 22Rv1 and PC3 cells exposed to Spautin-1 (20 μM) for the indicated duration

MAPKs in numerous cancers are well characterized as the main downstream signaling factors of EGFR to drive carcinogenesis and cancer progression [34, 35]. EGFR was also identified as an important target for treating PCa, especially for CPRC, which is insensitive to ADT [7, 8]. Given that Spautin-1 affects the function of MAPKs, we further asked whether Spautin-1 would inhibit the expression or activation of EGFR. Our western blot results showed that Spautin-1 notably decreased the levels of Tyr1068-phosphorylated EGFR and Tyr1173-phosphorylated EGFR but did not decrease total EGFR protein levels in 22Rv1 and PC3 cells (Fig. 5a), and these effects induced by Spautin-1 were paralleled with the effects of gefitinib, a classic EGFR-TKI (Fig. 5b), indicating that Spuatin-1 suppresses EGFR activation in prostate cancers. Activation of MKK4 is the precursory events of JNK activation, while phospho-MEK1/2 directly activates ERK1/2. To further investigate the early molecular events of EGFR signaling pathways induced by Spautin-1, we detected the expression of phospho-MKK4, MKK4, phospho-MEK1/2, MEK1/2, phospho-EGFR (Y1068), phospho-EGFR (Y1173) and total EGFR in PCa cells at different time points after initiation of Spautin-1 treatment. We found that Spautin-1 notably decreased the levels of EGFR phosphorylation at Tyr1068 and Tyr1173, as well as phospho-MEK1/2 from 2 h to 10 h, while activated MKK4 from 4 h to 8 h (Fig. 5c). Together, these results suggest that Spautin-1 inhibits the activation of EGFR and activates MKK4 and inhibits MEK1/2, thereby intervening its downstream pathways.

### Spautin-1 inhibited cell survival in glucose deprivation condition via down-regulating Glut1

**Fig. 6:**
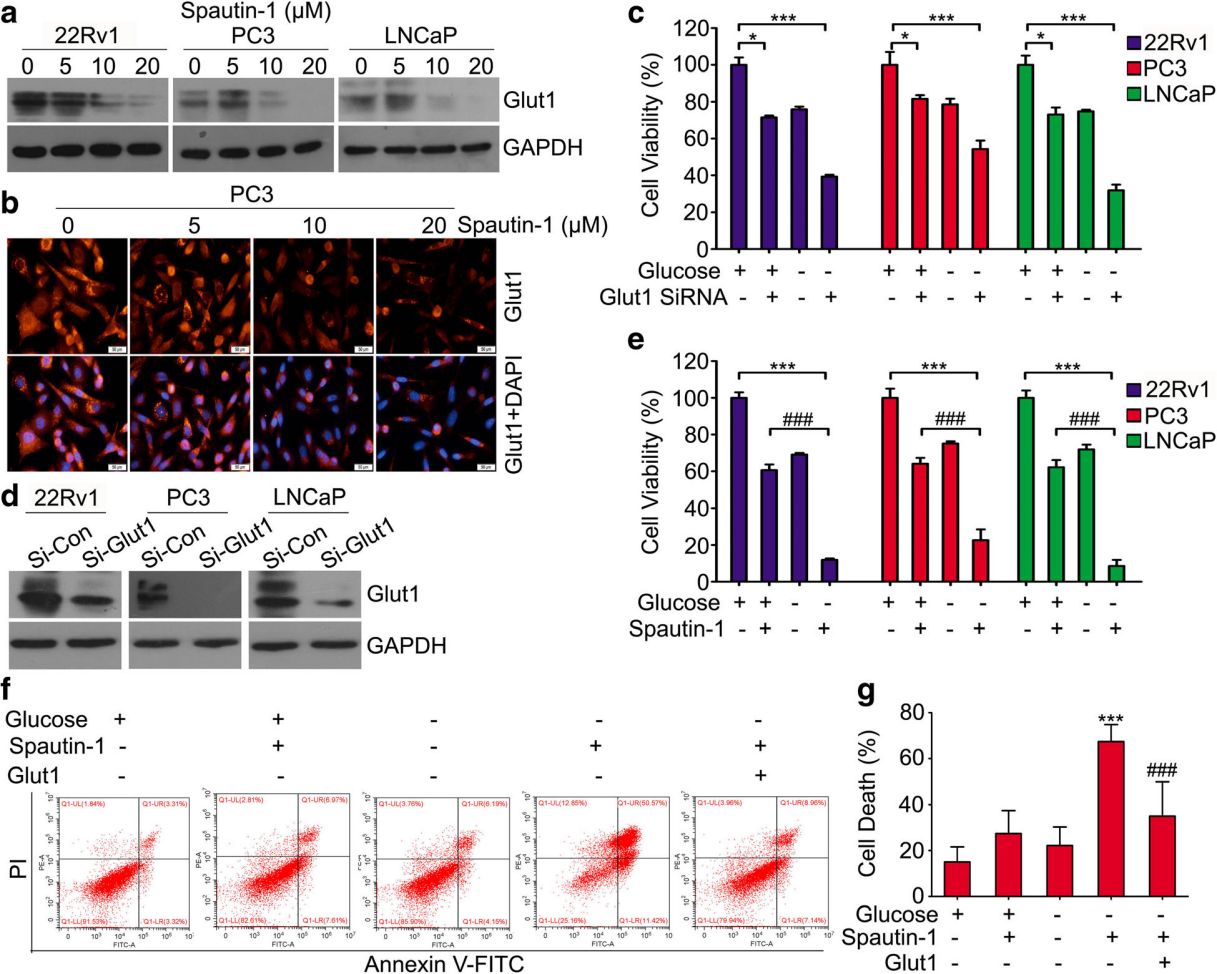
Spautin-1 inhibits cell survival in glucose deprivation condition via down-regulating Glut1. **a**) Western blot analysis and **b** immunofluorescence microscopy were performed to detect the expression of Glut1 in PCa cells treated with Spautin-1 for 24 h. **c** Cell viability assay was performed on PCa cells exposed to Glut1 siRNA or control siRNA for 48 h in the presence or absence of glucose. **d** Western blot analysis was performed to detect the expression of Glut1 in PCa cells exposed to Glut1 siRNA or control siRNA for 48 h. **e** Cell viability assay was performed on PCa cells exposed to Spautin-1 (10 μM) for 24 h in the presence or absence of glucose. **f** Cell death assay was performed on PC3 cells with or without Glut1 overexpression and exposed to Spautin-1 (10 μM) for 24 h in the presence or absence of glucose. Representative images from three independent experiments are shown. **g** Pooled data of **f** are shown. ***p < 0.001 vs. the glucose deprivation control group, ^###^*p* < 0.001 vs. the Spautin-1 combined with glucose deprivation group

Glut1 (glucose transporter 1) is crucial for glucose uptake and mediates multiple tumorigenesis and cancer progression [36–38]. Recent studies showed that Glut1 is overexpressed in PCa, possibly functions to support cell glycolysis and proliferation, and protects cells from oxidative damage induced by glucose deprivation [39, 40]. Interestingly, Glut1 is regulated by EGFR signaling. The activation of EGFR increases the expression of Glut1 [41, 42]. Hence, we investigated the effect of Spautin-1 treatment on Glut1 expression in PCa cells using western blot and immunofluorescence microscopy. We found that Spautin-1 notably decreased the expression of Glut1 (Fig. 6a, b and Additional file 1: Figure S4a). Subsequently, we investigated the effect of siRNA-mediated Glut1 KD on the cell proliferation of PCa cells in the absence or presence of glucose. We found that Glut1 KD decreased the cell viability of PCa cells in the presence of glucose, while more dramatically decreased the cell viability of PCa cells in the glucose deprivation condition (Fig. 6c and d). Since Glut1 is downregulated by Spautin-1, we then sought to test whether Spautin-1 would exhibit parallel effects to glucose starvation on PCa cells. We assessed the effect of Spautin-1 treatment on the cell viability of PCa cells in the absence or presence of glucose. We found that Spautin-1 showed more potently inhibitory effect on PCa cells in glucose deprivation than in normal condition (Fig. 6e). To further determine whether the potent proliferation inhibition effect induced by Spautin-1 combined with glucose deprivation is dependent on Glut1 expression or not, Glut1 recue experiment was performed on PC3 cells exposed to Spautin-1 in the presence or absence of glucose. It was found that Spautin-1 triggered more dramatic cell death of PC3 cells with glucose deprivation than in normal condition; however, this effect was significantly reversed by Glut1 overexpression (Fig. 6f and g). The effects of Glut1 overexpression were verified in Additional file 1: Figure S4b. These findings collectively suggest that Spautin-1 more effectively impairs PCa cells under glucose starvation, a situation that frequently occurs in the core of solid tumors, in a Glut1 dependent manner.

### Spautin-1 suppressed the growth of PCa xenografts

**Fig. 7:**
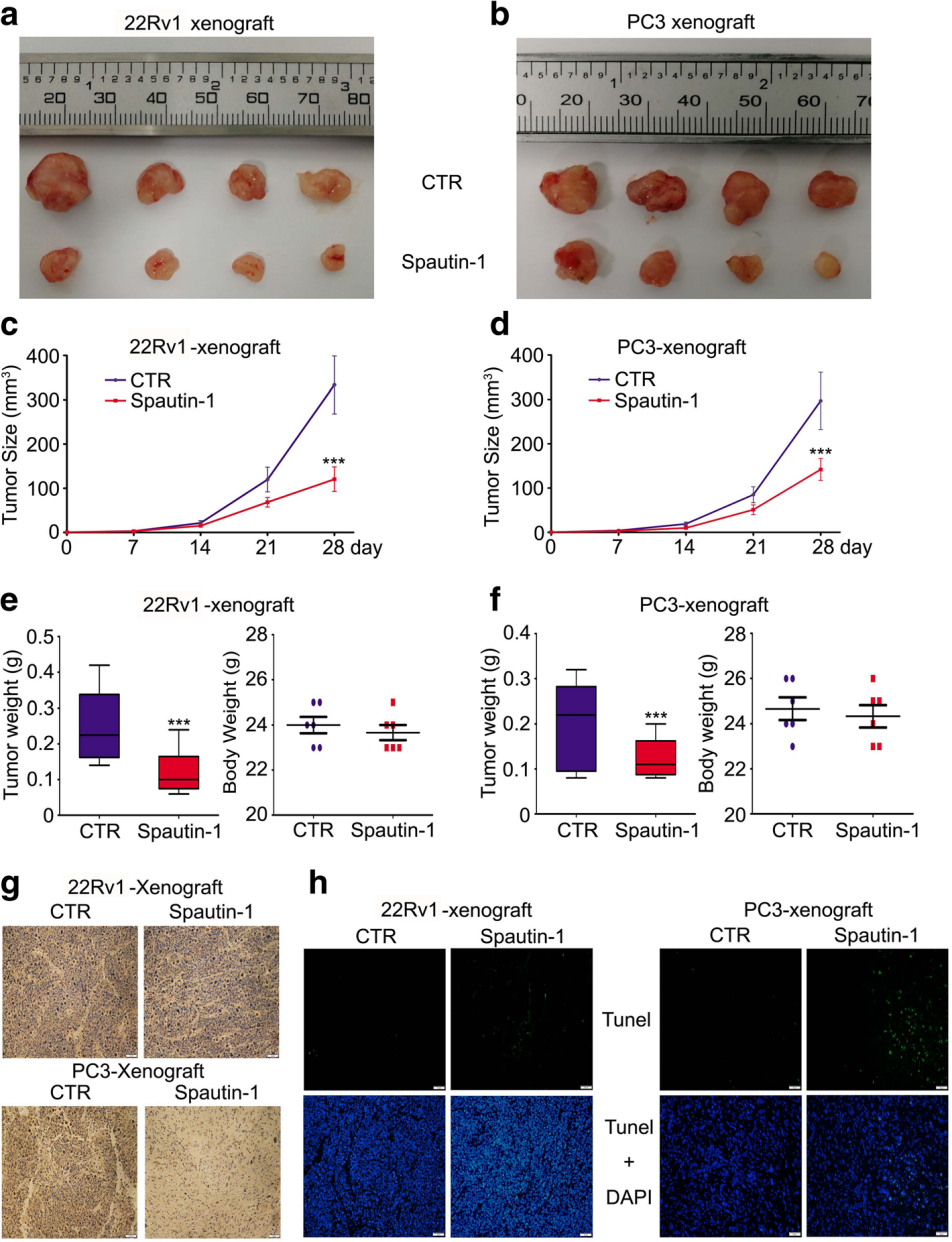
Spautin-1 suppresses PCa growth in vivo. **a** and **b** 22Rv1 and PC3 cells were engrafted into BALB/c nude mice and treated with Spautin-1 (20 mg/kg/d) for one month. Xenograft images are shown. **c** and **d** Tumor size was recorded weekly and summarized. **e** and **f** Tumor weight (left) and body weight (right) measured at the end of treatment are shown. ***p<0.05 versus vehicle control (CTR). **g** Immunohistochemistry staining assay was performed to detect the protein expression of Ki67 in the indicated xenograft samples. Representative images are shown. Scale bar = 50 μm. **h** TUNEL staining was performed to detect the apoptotic cells of xenografts. Representative images were shown. Scale bar = 50 μm

Nude mouse models were employed to further determine the in vivo effect of Spautin-1, a critical standard of testing anti-cancer compounds. As shown in Fig. 7a-d, the tumor size in the Spautin-1 treatment group was significantly decreased, compared with the control group. Additionally, tumor weights, but not body weights of mice, in the Spautin-1 treatment group were significantly reduced, compared with the control group (Fig. 7e and f). Our immunohistochemistry staining experiments showed that Spautin-1 substantially decreased the expression of Ki67 (Fig. 7g and Additional file 1: Figure S5a). Moreover, TUNEL staining assay showed that apoptotic cells in Spautin-1 treatment group were significantly increased compared with the control group (Fig. 7h and Additional file 1: Figure S5b). Collectively, these results indicate that Spautin-1 exhibits PCa suppression effects in vivo.

### Spautin-1 enhanced the sensitivity of PCa cells to enzalutamide in vitro and in vivo

**Fig. 8:**
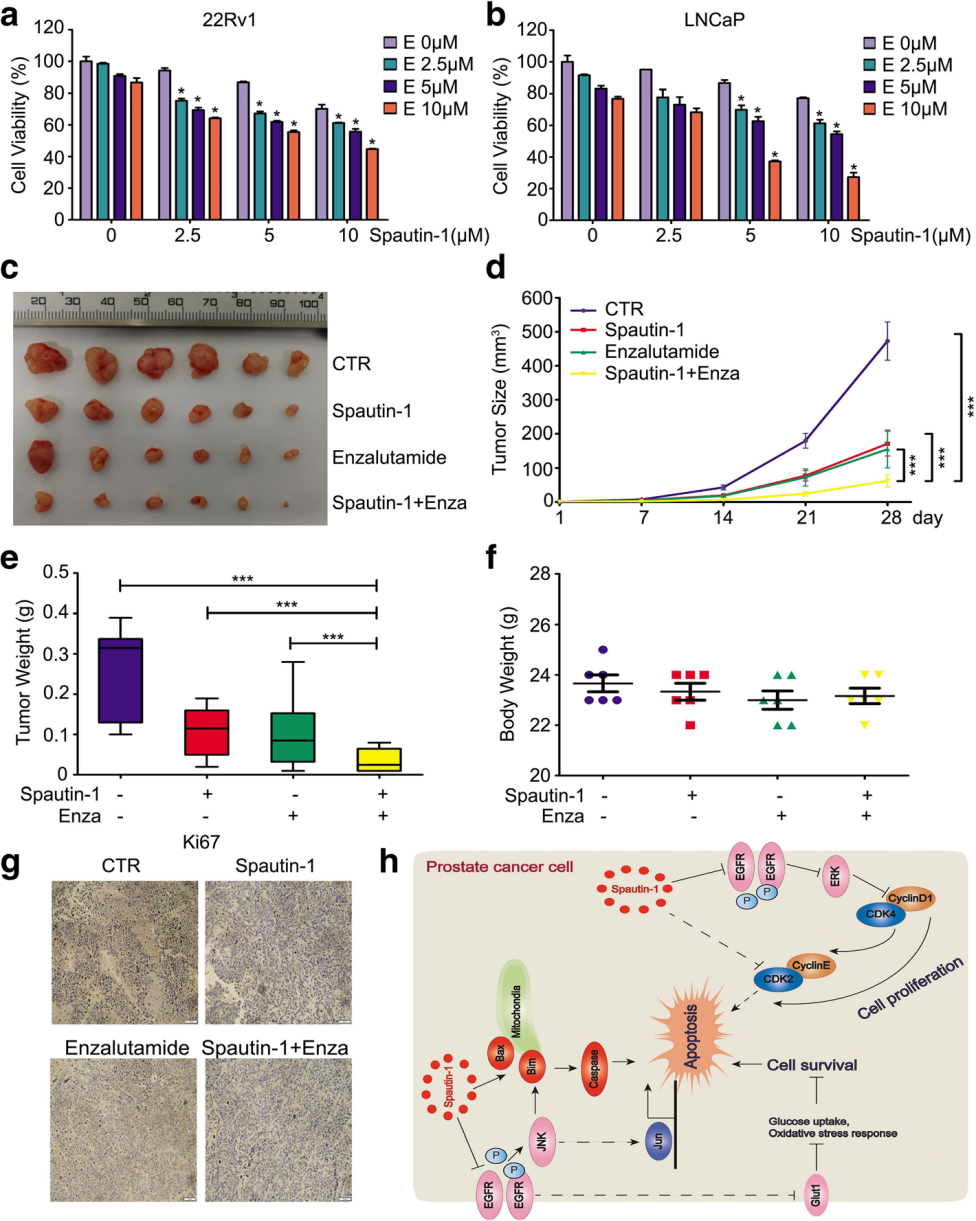
Spautin-1 enhances the sensitivity of PCa cells to Enzalutamide. **a**, **b** Cell viability assays were performed on PCa cells exposed to Spautin-1 in the presence or absence of Enzalutamide. *P<0.05 versus single drug treatment. **c**-**g** 22Rv1 cells were grafted into BALB/c nude mice and the mice were treated with Spautin-1 (20 mg/kg/d), Enzalutamide (25 mg/kg/d) or Spautin-1 in combination with Enzalutamide for one month. **c** Shown are xenograft images. **d** Tumor size, **e** tumor weight and **f** body weight are shown. *P<0.05. **g** Immunohistochemistry staining assay was performed to detect the protein expression of Ki67 of indicated xenografts. Representative images are shown. Scale bar = 50 μm. **h** A proposed model of Spautin-1 induced cell growth inhibition in PCa

Enzalutamide is the conventional agent approved by FDA for the treatment of advanced PCa. Spautin-1 showed a potent synergistic effect in combination with several anti-cancer agents. We further sought to address whether Spautin-1 enhances the anti-PCa effect of Enzalutamide. MTS assay was used to detect the cell viability of 22Rv1 and LNCaP cells treated with various concentrations of Spautin-1, Enzalutamide, or both. As presented in Fig. 8a and b, treatment combining Spautin-1 with Enzalutamide more remarkably reduced the cell viability of 22Rv1 and LNCaP cells than the treatment with Spautin-1 or Enzalutamide alone. We further investigated the in vivo effect of Spautin-1 in combination with Enzalutamide in nude mouse models. We found that tumor size and tumor weights in the combination treatment group were significantly reduced compared with the control, Spautin-1 or Enzalutamide alone treatment groups (Fig. 8c-f). Body weights of mice were comparable between the control and the various treatment groups. Moreover, immunohistochemistry staining assays showed that Ki67, an indicator of tumor proliferation, was also notably reduced in the combination group compared with the control, Spautin-1 or Enzalutamide alone treatment groups (Fig. 8g and Additional file 1: Figure S5b). These findings collectively indicate that Spautin-1 enhances the sensitivity of PCa cells to Enzalutamide both in vitro and in vivo, providing a potentially an alternative therapeutic strategy for the treatment of PCa.

## Discussion

Androgen receptor (AR) is the main driver of PCa development and progression. Androgen-AR binding triggers AR translocation from cytoplasm to the nucleus and activates the transcription of multiple androgen-responsive genes essential to supporting the proliferation and survival of PCa cells [43]. Traditional ADT aims to block the androgen-AR binding by diminishing androgens and is initially effective in PCa treatment. However, castration-resistance developed over time in most cases impedes its further application [44, 45]. Several mechanisms, including EGFR, intratumoral androgens, AR overexpression or mutation and AR cofactors, were proposed to contribute to AR signaling maintenance in an androgen-poor condition [43, 45, 46]. Increasing evidence shows the significance of EGFR in the progression of CRPC [7, 8, 47–49]; thus, inhibition of EGFR may represent a novel therapy to overcome CRPC.

In the current study, we found that Spautin-1 remarkably suppressed the proliferation of PCa cells, regardless of AR expression. Spautin-1 was primarily reported as an selective inhibitor of USP10/USP13 as well as autophagy inhibitor [16]. USP10 and USP13 are critical in the development of several cancers because they regulate some key tumor promoters or suppressors. Our previous study showed that Spautin-1 inhibits the growth of chronic myeloid leukemia via promoting the degradation of the oncoprotein, SKP2. Recent studies also have revealed that Spautin-1, alone or in combination with some classic anticancer agents, inhibits multiple cancers, such as ovarian cancer, osteosarcoma, chronic myeloid leukemia and colon cancer [17–20]. However, there is no definite mechanism by which Spautin-1 inhibits these cancers, possibly due to complexity of this chemical. Our further experiments showed that the proliferation inhibition effect induced by Spautin-1 was independent of USP10/USP13 and autophagy blockage. This phenomenon prompted us to address the underlying mechanisms. By the use of flow cytometry, we showed that Spautin-1 significantly induced G0/G1 phase arrest and cell death. Further investigation unraveled that Cyclin D1 is the key protein mediated Spautin-1 induced cell cycle arrest and that higher doses of Spautin-1 induced a caspase-dependent apoptosis in PCa.

MAPKs are critical to cell survival and proliferation in cancers. Mammals have three major groups of MAPKs: JNK, ERK and p38. Our findings indicate that Spautin-1 induces apoptosis through JNK activation and ERK inactivation in PCa cells. This is consistent with the general agreement that JNK activation can cause apoptosis while ERK activation contributes to cell survival [50]. In this study, we showed that the activation of JNK induced by Spautin-1 increased the expression of Bax and activated caspase-3, and eventually led to apoptosis. Additionally, activation of ERK was considered a driving force of G0/G1 cell cycle progression, possibly due to its induction of Cyclin D1 expression [51, 52]. Supporting the hypothesis that Spautin-1 induces cell cycle arrest through suppressing the ERK/Cyclin D1 axis, our further investigations showed that Spautin-1 activated MKK4 but not MKK7 (both the upstream kinase of JNK) and decreased the expression of phospho-MEK1/2 which directly controls the activation of ERK.

In general, MAPKs are highly regulated by EGFR. This study further explored the responsiveness of EGFR to Spautin-1 in PCa. We found that Spautin-1 treatment rapidly decreased the phosphorylation of EGFR, before the decrease of phospho-MEK1/2 and phospho-ERK1/2 and the increase of phospho-MKK4 and phospho-JNK became discernible. These findings indicate that the cell cycle arrest and apoptosis triggered by Spautin-1 through interfering MAPKs may root in the inhibition of EGFR. Inhibition of EGFR signaling decreases the expression of Glut1, possibly via decreasing the transcription and enhancing the degradation of Glut1, which was associated with ERK/PKM2/c-myc and AKT status [41, 42]. Therefore, Glut1 was regulated by EGFR in cancers. In the current study, we explored the effects of Spautin-1 on Glut1 expression in PCa on the basis of EGFR inhibition triggered by Spautin-1. We showed that Spautin-1 notably reduced the expression of the downstream effecter of EGFR, Glut1, that controls glucose uptake and cell survival in glucose deprivation condition. To combine previous studies and the current findings, we hypothesize that Spautin-1 induced Glut1 downregulation likely through EGFR inhibition. The downregulation of Glut1 induced by Spautin-1 notably enhanced cell death of PCa upon glucose removal, a state frequently occurs in the core of solid tumors [39].

Our in vivo experiments demonstrate that Spautin-1 has a potent inhibitory effect on the growth of PCa xenografts. The application of Spautin-1 did not apparently decrease the body weight of mice for one month, suggesting that Spautin-1 may have little undesired cytotoxicity toward normal tisues. Enzalutamide is the conventional anti-androgen agent for the treatment of advanced PCa. To explore whether Spautin-1 may enhance the efficacy of enzalutamide or even overcome enzalutamide resistance (or castration resistance) is significant for clinical translation of Spautin-1. The current study preliminarily try to address this question and hoping this combination therapy may benefit the patients with PCa in future. Therefore, further investigations were performed to determine the synergistic effects of Spautin-1 with Enzalutamide. Our results show that Spautin-1 notably sensitizes PCa cells to Enzalutamide both in vitro and in vivo. Hence, we suggest that Spautin-1 may serve as not only a standalone anticancer agent but a readily available adjuvant for Enzalutamide to treat PCa without obvious undesired cytotoxicity toward normal tissues.

## Conclusion

In summary, this study provides preclinical evidence that Spautin-1 inhibits EGFR signaling and thereby suppresses the growth of PCa. Inhibition of EGFR with Spautin-1 inactivates the MEK/ERK/Cyclin D1 axis and decreases Glut1 expression, while activating the MKK4/JNK/Bax axis, which collectively induced cell cycle arrest and apoptosis of PCa cells (Fig. 8h).

## Additional file

Additional file 1: **Figure S1.** Spautin-1 suppresses the proliferation of PCa independent of USP10 and USP13. **Figure S2.** High dose of Spautin-1 triggers caspase-dependent apoptosis in PCa cells. **Figure S3.** JNK and ERK mediate Spautin-1-induced growth inhibition. **Fig. S4** Spautin-1 inhibits cell survival in glucose deprivation condition via down-regulating Glut1. **Figure S5.** Spautin-1 suppresses PCa growth in vivo. (**a**) Immunohistochemistry staining assay was performed to detect the protein expression of Ki67 in the indicated xenograft samples. (DOCX 660 kb)

## Abbreviations

ADT: Androgen deprivation therapy
AR: Androgen receptor
CRPC: Castration-resistant PCa
EGFR: Epidermal growth factor receptor
Glut1: Glucose transporter 1
JNK: c-Jun N-terminal kinase
MAPK: Mitogen-activated protein kinase
Pca: Prostate cancer
PI3K: Phosphoinositide-3-kinase
USP10: Ubiquitin-specific peptidase 10
