# Supplementary material for: Inhibition of EGFR signaling with Spautin-1 represents a novel therapeutics for prostate cancer
Source: J Exp Clin Cancer Res. 2019 Apr 11;38:157. doi: 10.1186/s13046-019-1165-4 (PMC6460657; doi:10.1186/s13046-019-1165-4)
Supplement: Supplementary file 1 — Figure S1. Spautin-1 suppresses the proliferation of PCa independent of USP10 and USP13. Figure S2. High dose of Spautin-1 triggers caspase-dependent apoptosis in PCa cells. Figure S3. JNK and ERK mediate Spautin-1-induced growth inhibition. Fig. S4 Spautin-1 inhibits cell survival in glucose deprivation condition via down-regulating Glut1. Figure S5. Spautin-1 suppresses PCa growth in vivo. (a) Immunohistochemistry staining assay was performed to detect the protein expression of Ki67 in the indicated xenograft samples. (DOCX 660 kb) [file 13046_2019_1165_MOESM1_ESM.docx]

**
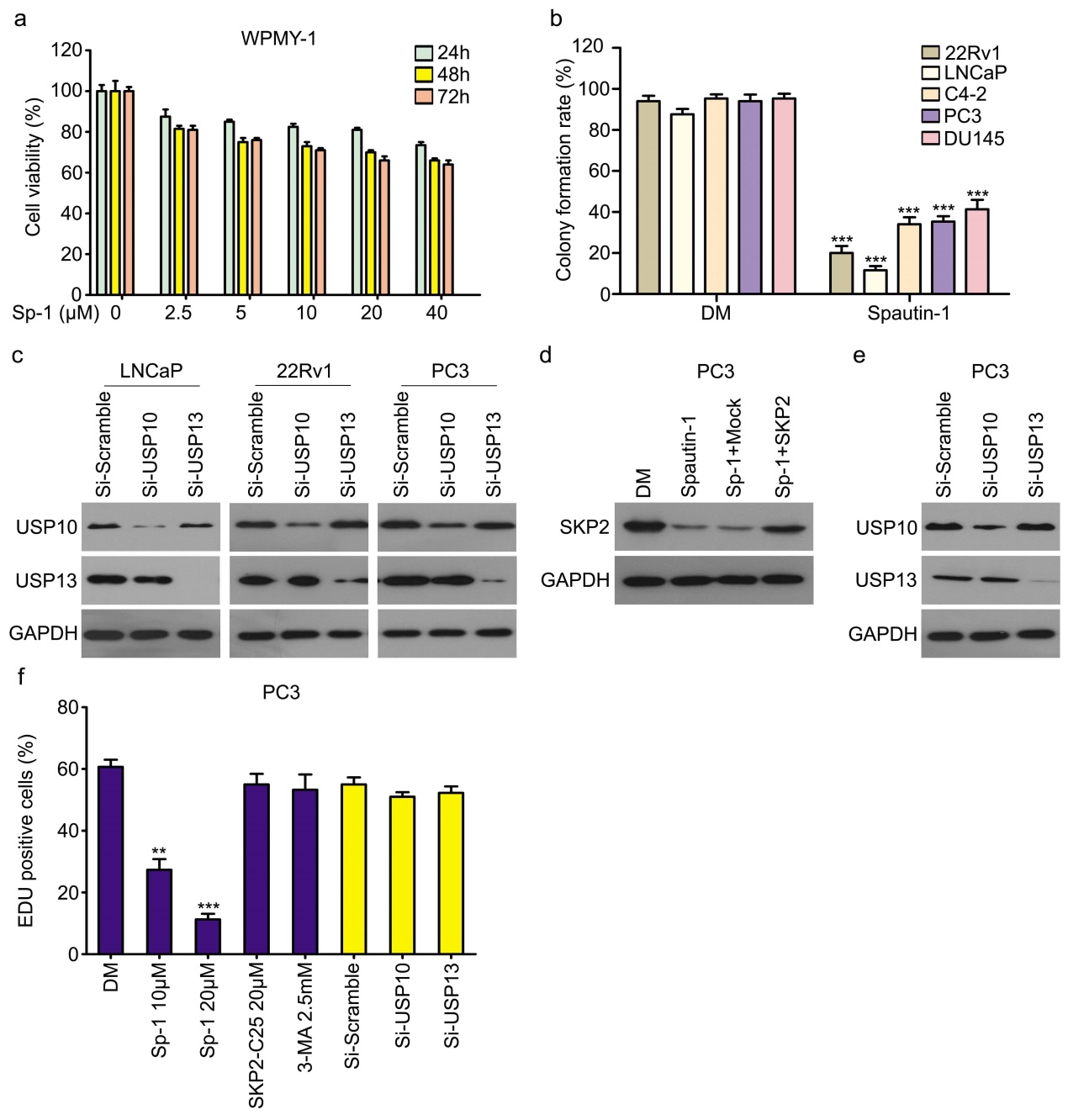
**

**Fig. S1** Spautin-1 suppresses the proliferation of PCa independent of USP10 and USP13. (**a**) Cell viability assay was performed in WPMY-1 cells post various concentrations of Spautin-1 treatment for indicated hours. (**b**) Colony formation assay was performed in PCa cells post Spautin-1 (10 μM) treatment for two weeks. Data quantification were shown. DM, DMSO. ^***^*P* < 0.001. (**c**) Western blot of USP10 and USP13 to verify the efficacy of USP10 and USP13 KD in prostate cancer cells. (**d**) Western blot was performed to detect SKP2 level in PC3 cells pre-transfected with control vector or SKP2 plasmid for 24 h before Spautin-1 (20 μM) treatment for 48 h. (**e**) Western blot of USP10 and USP13 to verify the efficacy of USP10 and USP13 KD in PC3 cells. (**f**) Edu staining assay was performed to detect the proliferation ability of PC3 cells treated with Spautin-1, SKP2-C25, or 3-MA treatment for 24 h, or 48 h after subject to USP10 knockdown (KD) and USP13 KD. Data quantification were shown. DM, DMSO. Sp-1: Spautin-1. ^**^*P* < 0.01; ^***^*P* < 0.001.


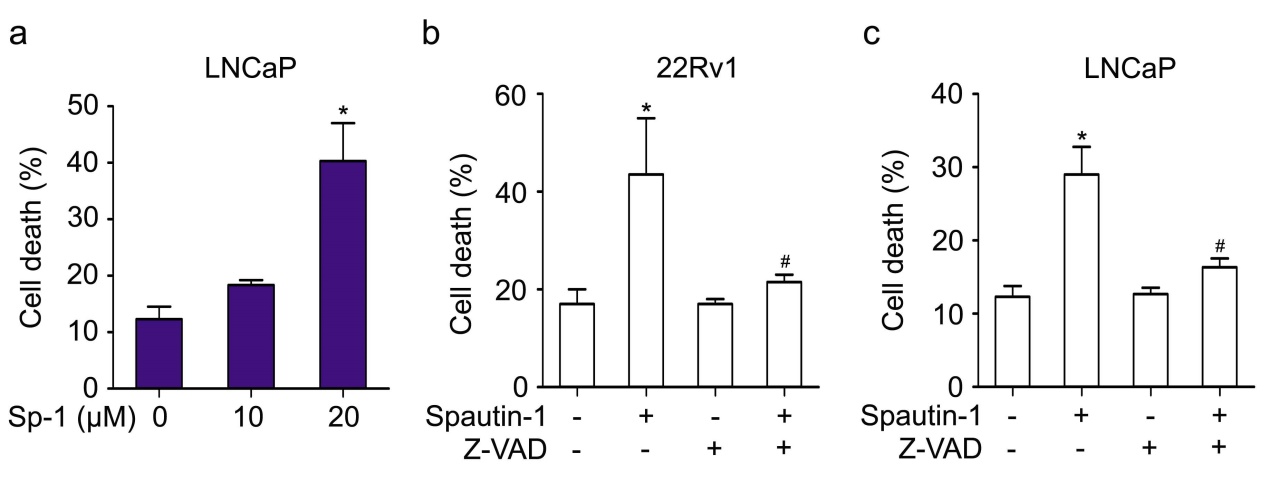


**Fig. S2** High dose of Spautin-1 triggers caspase-dependent apoptosis in PCa cells. (**a**) Flow cytometry analysis following Annexin V-FITC and PI staining was performed on LNCaP cells exposed to Spautin-1 for 24 h. Data of three independent repeats are summarized. Sp-1: Spautin-1. ^*^*P*﹤0.05 *versus* control treatment. (**b**) and (**c**) Apoptosis assay was performed on 22Rv1 and LNCaP cells exposed to Spautin-1 (20 μM) in the presence or absence of Z-VAD-FMK (50 μM). Shown are pooled data from three independent experiments. ^*^*P*﹤0.05 *versus* control treatment; ^#^*P*﹤0.05 *versus* Spautin-1 treatment.

**
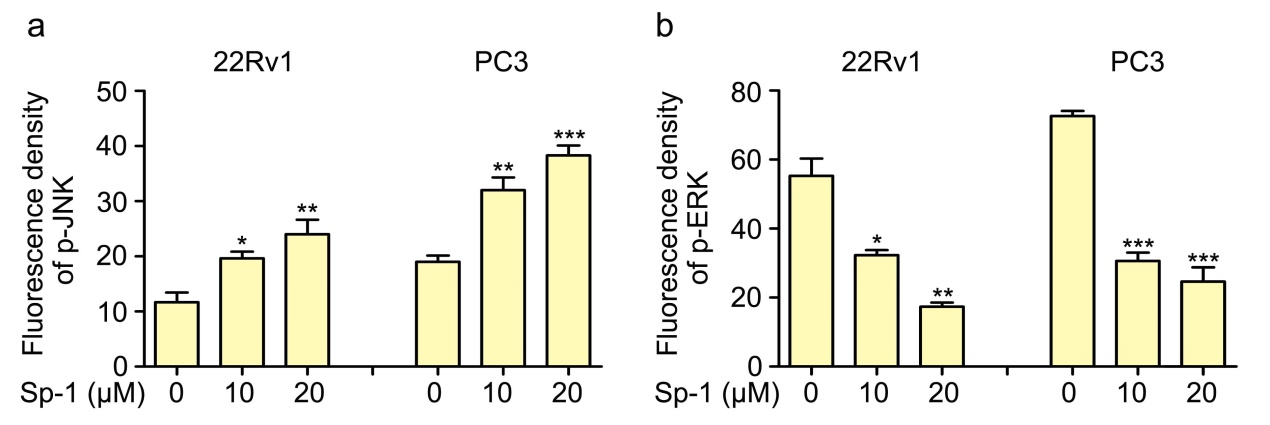
**

**Fig. S3.** JNK and ERK mediate Spautin-1-induced growth inhibition. (**a**) and (**b**) Immunofluorescence microscopy was performed to detect the expression and subcellular location of phospho-JNK and phospho-ERK in 22Rv1 and PC3 cells exposed to Spautin-1 for 6 h. Shown are pooled data from three independent experiments. Sp-1: Spautin-1. ^*^*P*﹤0.05; ^**^*P* < 0.01; ^***^*P* < 0.001.

**
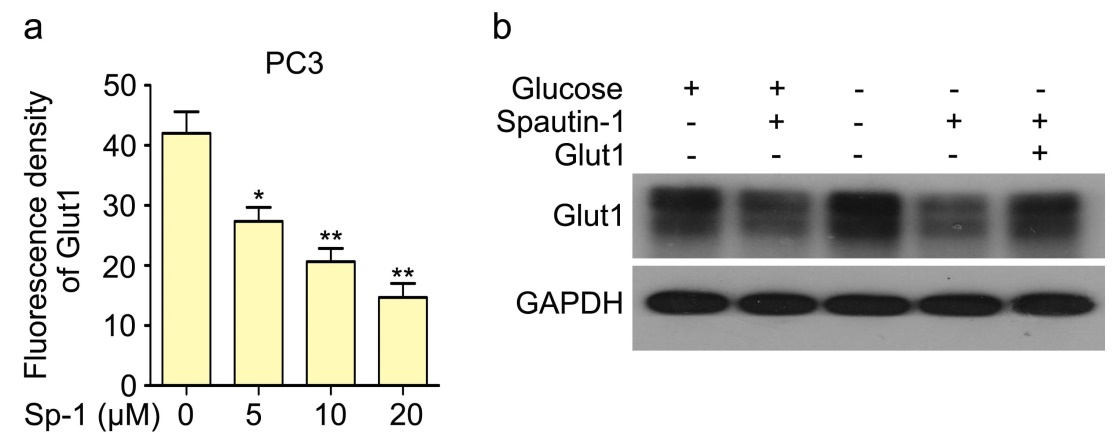
**

**Fig. S4.** Spautin-1 inhibits cell survival in glucose deprivation condition *via* down-regulating Glut1. (**a**) Immunofluorescence microscopy were performed to detect the expression of Glut1 in PCa cells treated with Spautin-1 for 24 h. Shown are pooled data from three independent experiments. Sp-1: Spautin-1.^*^*P*﹤0.05; ^**^*P* < 0.01. (**b**) Western blot was performed to verify the expression of Glut1 on PC3 cells with or without Glut1 overexpression and exposed to Spautin-1 (10 μM) for 24 h in the presence or absence of glucose.

**
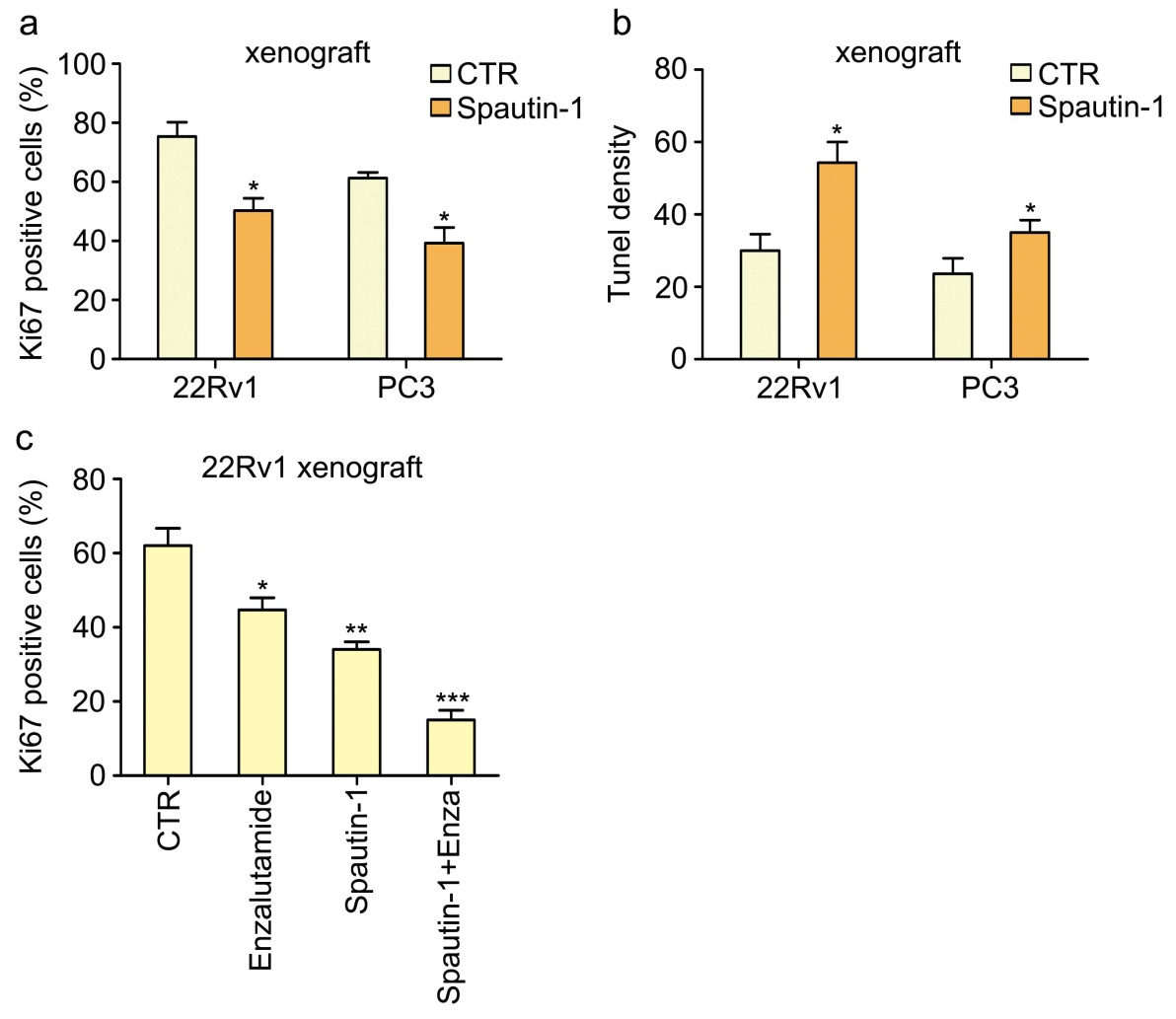
**

**Fig. S5.** Spautin-1 suppresses PCa growth *in vivo*. (**a**) Immunohistochemistry staining assay was performed to detect the protein expression of Ki67 in the indicated xenograft samples. Data quantification were shown. ^*^*P*﹤0.05. (**b**) TUNEL staining was performed to detect the apoptotic cells of xenografts. Data quantification were shown. ^*^*P*﹤0.05. (**c**) Immunohistochemistry staining assay was performed to detect the protein expression of Ki67 in the indicated 22Rv1 xenograft samples. Data quantification were shown. ^*^*P*﹤0.05; ^**^*P* < 0.01.
